# The Role of Play in Children’s Palliative Care

**DOI:** 10.3390/children1030302

**Published:** 2014-10-01

**Authors:** Sue Boucher, Julia Downing, Rise Shemilt

**Affiliations:** 1 International Children’s Palliative Care Network (ICPCN), Assagay 3610, South Africa; Shuter & Shooter Publishers (Pty) Ltd, Pietermaritzburg, 3212, South Africa; E-mail: sue@icpcn.co.za; 2 International Children’s Palliative Care Network, Assagay, 3610, South Africa; Makerere University, Kampala, Uganda; E-Mail: Julia.downing792@btinternet.com; 3 Paediatric Palliative Care Service, Gaddum Centre, Manchester, M15 4AX, United Kingdom; E-Mail: rshemilt@gaddumcentre.co.uk

**Keywords:** children, role of play, value of play, life limiting illness, life threatening illness, psychological wellbeing

## Abstract

Play is the universal language of childhood and the time and opportunity to play is every child’s right. The role of play as a vehicle for communication, a tool for distraction and its value in the holistic development of a normal child is without dispute. The role and value of play increases proportionately when a child is made more vulnerable through illness or disability. Despite this, providing time and opportunities to play can be overlooked or considered to be of little importance or relevance when the focus of the adult carers is the amelioration of clinical symptoms of the illness and on lessening the psychological impact the illness may have on the child. This paper outlines the role and the value of play as an integral component in the provision of palliative care for children with chronic, life-threatening and life-limiting conditions. It will show how providing appropriate equipment, sufficient time and relevant play opportunities not only improves the very sick child’s psychological wellbeing, but also allows the child to cast aside the confines and restrictions imposed upon them by their illness and for a few golden moments to be nothing more than a child at play.

## 1. Introduction

“*The sky’s awake so I’m awake and I have to play*”.

(Princess Anna from Disney’s *Frozen*)

It is generally agreed that play is an important part of children’s development, education and learning, and that play develops with the mind, and the mind develops with play [1,2,3,4,5,6]. Play is the language of children. Play is the “work” of children. Play is the way that children learn about their world, become competent at skills, learn to cooperate with others and gain mastery over their emotions. Play is what motivates children to get out of bed in the morning. Think of children and one thinks of play. Mention toys and one thinks of children. However, thoughts of very sick children or children with severe limitations or restrictions on their mobility, their stamina or their cognitive capacity do not as quickly turn to thoughts of toys and play. As responsible adults our overwhelming impulse when faced with a very sick and vulnerable child is to find ways to improve that child’s health, take away distressing symptoms and to provide the most appropriate medications and therapies. It does not sit well with us that children suffer and die, but they do. It is estimated that globally around 18,000 children under the age of 5 years will die each day, a figure which equates to around 11 million children dying every year [7].

While more than 50% of these deaths are from preventable causes [7] there are still millions of children who will die this year or whose lives are threatened by diseases such as cancer, heart disease and genetic conditions. These children live with conditions which will shorten their lives and lead to their death either in childhood or early adulthood [8]. While still not universally accessible [9,10], all children with life-threatening and life-limiting conditions have the right to receive palliative care which aims to ameliorate the unpleasant symptoms of the illness or condition and improve the quality of life of the sick child and that child’s family [10,11,12,13].

When a child is diagnosed with an illness or condition likely to considerably shorten their lifespan, there are many challenges as well as new and frightening experiences that both the child and the child’s family will inevitably face. These experiences can and often do overwhelm the child, the parents and family members and all those close to the family. The provision of palliative care from the moment of diagnosis allows the “breathing space” for those who care most for the child to adjust and to continually readjust to their new reality.

“*When my son was diagnosed I felt as if all my self-esteem had disappeared. I could not see my future, and I felt like my family was a freak show for everybody to look at. All I could think about was his death and thoughts of this preoccupied everything I did*”*.*

[14] (p.7)

These words of a mother whose child was diagnosed with a life-limiting illness illustrate this feeling of devastation and loss of control. Palliative care providers walk the journey from diagnoses, through to the end-of-life and into bereavement along with the child and the child’s family, providing appropriate and compassionate support whenever and wherever it is needed. However, despite the devastating consequences of a terminal diagnosis, the child continues to have all the unique and ever-evolving needs, desires and the rights of any other child [15]. The overwhelming urge and desire to play still exists, indeed, it could be argued that because of the benefits of play, these children have an even greater need to play than other children [6] as can be seen in the case study in Box 1.

**Box 1. Case Study.**

*Sunflower House Children’s Hospice in Bloemfontein, South Africa is seldom without the sound of children playing and their noisy chatter and laughter. While the children cared for at this hospice all have an illness or condition that threatens or will likely shorten their lives, on this warm afternoon in December 2008 most looked remarkably robust and were engaged in exuberant play, both in and out of doors. I came across Lebo lying in a small, quiet room with just one bed. It was a room, I was told, often used by the sickest children—those whose deteriorating health prevented them from coping with the normal day-to-day activities of the hospice. It was also used at times for those nearing the end of life. Almost 7 years old, Lebo looked closer to 4; his stunted growth the result of congenital heart disease. His energy levels were depleted despite the oxygen being fed to him via a saturator. Any pain he had was well controlled but there was no mistaking the anxiety and sadness in his eyes as they followed me into the room. We did not share a common language but no words were needed for me to know that he would rather be playing with his friends. While he had managed to tear the paper wrapping off a gift he’d been given to reveal a plastic fishing rod with a handful of colourful fish, the stiff packaging had proven too much for Lebo so the toy lay still unopened on the bed. Releasing the toy for him and without words we began to play together. I pretended to be completely inept at catching the fish which made him smile. I clapped whenever he caught a fish, which delighted him even more. I hid the fish in the folds of the bedclothes and he found them again to raspy giggles. For a while we played together and Lebo smiled and laughed and clapped in delight. When I had to leave I was rewarded with the sweetest smile and a wave. Lebo died not long after my visit, an event not unexpected as he had fought to live almost every day of his short and life and had faced many difficulties, both social, emotional and physical. But for a few golden moments of time on that warm December day Lebo was able to be nothing more, and most certainly nothing less, than a child at play.*

## 2. Children’s Rights and Play

Article 23 of the UN Convention on the rights of the child [16] calls upon all parties to *recognise that a mentally or physically disabled child should enjoy a full and decent life, in conditions which ensure dignity, promote self-reliance and facilitate the child's active participation in the community* (p. 8)*.* Article 31 of the same convention calls upon all parties to recognise the right of the child to rest and leisure, to engage in play and recreational activities appropriate to the age of the child and to participate freely in cultural life and the arts. Article 31 also calls upon all parties to respect and promote the right of the child to participate freely in cultural and artistic life and *encourage the provision of appropriate and equal opportunities for cultural, artistic, recreational and leisure activity* (p. 10).

The International Children’s Palliative Care Network (ICPN), the only organisation working globally to champion the rights of life limited children, published a charter of rights of life limited and life threatened children [17]. The Charter states that, wherever possible, life limited and life threatened children be provided with opportunities to play, access leisure opportunities, interact with siblings and friends and participate in normal childhood activities.

## 3. Palliative Care for Children

Palliative care aims to optimise quality of life in the face of an ultimately terminal condition. The World Health Organization (WHO) defines palliative care for children as “*the active total care of the child’s body*,* mind and spirit*,* (which) also involves giving support to the family. It begins when illness is diagnosed*,* and continues regardless of whether or not a child receives treatment directed at the disease. Health providers must evaluate and alleviate a child’s physical*,* psychological*,* and social distress*” [18]. Palliative care is not a single intervention; it is a philosophy of care. It can be seen as a thread that weaves through the lives of all children with a life limiting illness, often alongside active interventions and treatment.

Palliative care for children is appropriate from the time of diagnosis, even when there is hope for a cure; it continues throughout the trajectory of the illness and, should no cure be possible, supports the child and family through death and into the bereavement period for as long as it is needed [18]. Children and young people with life-limiting or life-threatening conditions have distinctive palliative care needs, which differ significantly from those of adults [15,19]. For example, communication with children is often more challenging and their understanding of death and dying is not the same as that of adults [15].

Ideally, this care is provided by a multi-disciplinary team of professionals that could include doctors, nurses, psychologists, social workers, counsellors, physiotherapists, play therapists, music therapists, educators and any others, according to the specific and individual needs of each child and the resources available [20]. Members of this multi-disciplinary team undergo specific training in order to be effective in lessening any physical, social, psychological, emotional, cognitive or spiritual suffering the child and family members’ may experience.

### 3.1. Where is Palliative Care for Children Provided?

Palliative care for children can be delivered in a diversity of settings, including a hospital, an out-patient clinic, an in-patient care unit, a purpose built children’s hospice, a community centre, or in the child’s own home [18]. The important thing in the provision of palliative care for children is that palliative care is a philosophy of care that should be provided wherever the child and their family are being cared for, and the model of care delivery will vary according to culture, resources and family preferences.

### 3.2. Eligibility Criteria

Eligibility criteria for children’s palliative care differs widely according to the specific service required and geography. The stage of the illness will determine what level of palliative care is required while geography influences who is eligible to access available services. For example, in sub-Saharan Africa, most children accessing palliative care services have HIV and/or cancer [21,22], and the same is true for adults; in the United Kingdom, however, the breadth of the disease profile of children who can receive these services is expansive and very different from that of adults. Some children will need respite care and some long-term access to services, while others will have only a few days to live [23].

In order to help identify children in need of palliative care, the Association for Children’s Palliative Care (now called Together for Short Lives) and the Royal College of Paediatrics and Child Health developed categories of children with life limiting and life-threatening illnesses [24,25]. The categories act as a guide, but the most important factor is not identifying which category fits a child but ensuring that the child has access to palliative care [22]. The four categories are: (1) life-threatening conditions for which curative treatment may be feasible but could fail; (2) conditions for which there may be long phases of intensive treatment aimed at prolonging life, but premature death is still possible; (3) progressive conditions for which there is no curative treatment; and (4) conditions that are not considered progressive but are characterised by severe neurological disability, which may cause the patient to deteriorate unpredictably [24].

## 4. The Effect of a Life-Limiting Illness on a Child

The effects of any chronic illness are likely to be pervasive, particularly if the illness threatens or will limit the life of the child. There is likely to be a significant amount of stress and anxiety experienced by the child. In addition, limitations due to illness often place obstacles in the way of normal development.

Apart from the very obvious physical effects that any chronic or life limiting illness has on the child, Goodman [26] lists common psychological problems experienced by children with chronic and life limiting illnesses to include the internalisation of problems leading to anxiety, depression, fear, hopelessness, helplessness, loss of control and frustration; the externalisation of problems which leads to aggression, noncompliance and withdrawal; somatic complaints such as pain and impaired functioning; self-concept issues manifesting in poor self-image and low self-esteem and social and educational difficulties such as academic and learning problems and decreased or deficient social competence.

Particular problems will be associated across different illnesses and for different reasons. For example, cognitive deficits are a possible complication for children who have had cranial radiation and for children who have had strokes.

How a child responds and ultimately adjusts to a diagnosis will differ according to a variety of factors, including the type of illness and the effect of the treatment on the child, the child’s age, mental capacity and personality. Other factors influencing a child’s response include:

- The degree to which the illness impairs the child’s ability to continue with normal activities and functioning, e.g., a child with cancer may have sporadic school attendance thus impacting academic progress.
- The involvement of the brain, e.g., if there are learning problems and loss of social skills.
- The severity and the course of the illness.
- The child’s view of death—as the illness progresses their fear of death may heighten
- The type of medical procedures and their experience in hospital
- The way the family functions and the effect of the illness on the family will influence the child. The child copes best when a family is cohesive, flexible, supportive, and communication is open and clear.
- The internal resources of the child pre-illness, for example how they cope in stressful situations.
- External resources and support systems: adequate outside support can have a positive effect on the child and family's adjustment and is related to personal, financial and geographic factors [26].

## 5. The Needs of the Life-Limited Child

“*Food, toys and love are what we need to live*.”.

(4 year old child) [27] (p. 112)

“*If kids are normal, not sick, they like to be treated special. But if kids have a disease, they wanted to be treated normal*.”.

(11 year old girl) [27] (p. 82)

The second of these two statements from children, recorded in the book “Armfuls of Time” [28] reflects the duality experienced by children with a chronic or life-limiting condition. Their longing for a normal life and to be treated as “normal” children while simultaneously coming to terms with the “abnormal” circumstances their illness or conditions have thrust upon them and their families. Sourkes makes the point that parents are faced with the difficult task of providing all the care and support required by the illness while making every effort to keep the child’s life as normal as possible, which at times still includes the need for discipline and boundary setting. It could mean finding a way for the sick child to attend school whenever possible or providing opportunities for them to socialise with their friends. This, says Sourkes, fosters self-esteem and communicates a critical message to the child that while the illness may be abnormal, the child remains normal in their eyes [28].

Love, respect and dignity are just as important for children with a life limiting illness as for adults [29]. The University of Rochester Medical Center [29] suggests that the dying child has specific psychosocial needs which include: allowing the child to partake in age-appropriate activities and play; allowing the child to communicate openly and honestly, to express their fears and anger and to be listened to in a non-judgemental manner; ensuring the child has as much independence and control as is possible within the limits of their illness whilst respecting boundaries and age-appropriate limits; respecting the child’s spiritual needs and allowing them to express these as appropriate according to their culture and background; and the need for reassurance that they are not alone, and that they will be missed by their loved ones when they die.

In their book on developmental and spiritual issues of dying children, Jones and Weisenfluh [30] contend that children facing life-limiting and life-threatening conditions have unique psychosocial and spiritual needs and those who provide care need to create interventions and models of support that will meet these needs. They make the point that after reaching a certain age or cognitive level children have the capacity for autonomy, choice, and thoughtful decision-making and should be included throughout the treatment process and in end-of-life decisions. At the same time, clinicians caring for dying children should remember that they continue to be children throughout the illness and dying process. Consequently it is important for these professionals to maintain an understanding of the developmental stages of children as defined by Piaget [31] and how these will in turn interact with the illness and the dying process.

Jones and Weisenfluh [30] state that one of the greatest needs of children with a life limiting illness is for those providing care to be honest and to tell them exactly what is happening to them. This approach allows the dying child a modicum of control and the opportunity to express their own choices. Questions the children may have include “*Will you come with me?*” “*What does heaven look like?*” “*Can I bring my toys?*” “*Will my mom and dad be ok?*” “*Will it hurt?*” “*Who is God?*” “*Will you remember me?*”

To be able to deal with these questions children need to have people caring for them who have a firm grasp of a child’s cognitive abilities and of developmental stages in order to contextualise and provide appropriate responses to the questions they ask and concerns that they raise.

In summary, the dying child needs to participate in age appropriate activities that allow them to “just be children” while providing opportunities for them to experience a level of independence and control. They need to be provided with opportunities and the means by which to express their fears and anxieties and to communicate their questions and concerns about their own death in a non-threatening environment with carers who will respond appropriately. They need to participate in activities that will allow them to continue along physical, social, emotional and cognitive developmental pathways in order for them to reach their full potential, and despite their illness, they still need to be given boundaries, to learn self-control and how to best inhabit their own humanity.

## 6. The Role of Play in the Provision of Children’s Palliative Care

### 6.1. What is Play?

Definitions on play abound, each of them differing in accordance with the primary focus of the definer. In the General comment No. 17 on Article 31 of the UN Convention on the Rights of the Child [32], the legal definition of play is recorded as:

“*behaviour, activity, or processes initiated, controlled and structured by children themselves and it takes place whenever and wherever opportunities arise. Caregivers may contribute to the creation of environments in which it takes place, but play itself is non-compulsory, driven by intrinsic motivation and is undertaken for its own sake, rather than as a means to an end. It involves the exercise of autonomy, physical, mental or emotional activity, and has the potential to take infinite forms, either in groups or alone. These forms will change and be adapted throughout the course of childhood. The key characteristics of play are fun, uncertainty, challenge, flexibility and non-productivity. Together, these factors contribute to the enjoyment it produces and the consequent incentive to continue to play. While play is often considered non-essential, the Committee reaffirms that it is a fundamental and vital dimension of the pleasure of childhood, as well as an essential component of physical, social, cognitive, emotional and spiritual development.*”.

[32] (pp. 5–6)

Gray [33], defines play as a confluence of several characteristics which he distils to the following five:

1. *Play is self-chosen and self-directed; players are always free to quit.* Play is therefore what one *wants* to do at that moment and gives a feeling of liberty. Grey also makes the point that when adults play with children they should resist the urge to control the play as children may feel obliged to continue even when they do not wish to do so.
2. *Play is activity in which means are more valued than* *end*. The focus on the process and not the product of any play activity effectively removes any fear of failure. This also gives the player the freedom to experiment with new ways of doing things.
3. *Play has structure, or rules, which are not dictated by physical necessity but emanate from the minds of the players.* The child is willing to accept the rules of the game only because at any moment, he or she has the freedom to choose to leave it. In agreement with Vygotsky, who makes the point that the child’s desire to play is so strong it becomes the motivating force for self-control [34], Grey contends that “*the greatest of play’s many values for our species lies in the learning of self-control… Everywhere*,* to live in human society*,* people must behave in accordance with conscious*,* shared mental conceptions of what is appropriate*;* and that is what children practice constantly in their play. In play, from their own desires*,* children practice the art of being human*.”
4. *Play is imaginative*, *non-literal, mentally removed in some way from “real” or “serious” life. It is* “*serious yet not serious*,* real yet not real*.* In play one enters a realm that is physically located in the real world*,* makes use of props in the real world*,* is often about the real world*,* is said by the players to be real, and yet in some way is mentally removed from the real world.”* The role of fantasy and imagination is central to most forms of play and is governed by rules within the minds of the players and not by the laws of nature. For example a broom can be a horse or, as in the game of chess, a particular shaped piece of wood can be a castle. Thus through play the child learns to take charge of the world and not simply respond passively to it. Children are also able to switch from being completely absorbed in the fantasy of the game, to returning to reality as needed—they are able to distinguish between real and pretend yet at times prefer to stay in the “magic” of the game as opposed to re-entering the “real world”.
5. *Play involves an active*,* alert*,* but non-stressed frame of mind.* Grey asserts that this last characteristic follows naturally from the other four. Because of the very nature of play, the player is freed from the “*strong drives and emotions that are experienced as pressure or stress.*” Some refer to this state of play as “*flow*” where attention is so attuned to the activity, there is reduced consciousness of self and time.

In Grey’s opinion [33], the more fully an activity entails all of these characteristics, the more inclined most people are to refer to that activity as play, including the players themselves. He also notes that all of the characteristics describe the motivation or attitude that the player brings to the activity, rather than the activity itself.

Play is central to the life of the young child. It occupies most of his time between daily routines. It contributes to every single aspect of his development and lays the foundation for almost everything that he learns before he goes to school. Play is the means by which the child explores and masters his world and the non-verbal expression of his experience of reality [6].

### 6.2. Understanding the Needs of the Dying Child through Play

“*Whoever wants to understand much must play much*.”.

*Gottfried Benn* [35]

In her paper on the use of play therapy in paediatric palliative care, van Breeman tells us that in order to provide comprehensive care for the dying child, nurses can expand their role and offer support to the parents or caregivers, listen and reflect the child’s hopes and fears expressed through the child’s play, and facilitate the development of positive family relationships and healing during the dying journey [36].

van Breeman refers to McDonald and McIntyre’s [37] assertion that the manner in which individuals gain knowledge and a sense of value from their lives is through the experiences of the body and the interpretation of those experiences by the mind. In a similar way to adults, children also seek to make sense of their experiences in living and dying. There is a widely held belief in palliative care that patients should be considered the ultimate experts on their own lives. van Breeman concludes that this includes children, who, regardless of their age, need to be given opportunities to share their history and incorporate it into their dying journey [36].

One of the most effective ways to allow this history telling is for those providing care to a dying patient to take the time to listen to their narrative. This provides the insight needed to experience what the person is feeling, how they want to be treated and how much they are ready to hear. These narratives are usually told via the spoken or written word. Children will however differ in their ability to use language to tell their story, due to their illness and/or stage of development, thus utilising play instead [36].

Play provides children with a safe and acceptable way to deal with the fallout from traumatic experiences. Trauma is “*an impingement that occurs before the individual has developed mechanisms that make the unpredictable, predictable*” [38] (p. 151). The devastating impact of a life threatening illness on the child qualifies as trauma. Winnicott, the first British paediatrician to become a psychoanalyst, stressed the importance for the young child of the preservation of a certain amount of illusion, and avoiding a sudden insistence on reality [39]. The child with a life-threatening illness faces the ultimate threat of loss of life and it is fair to say that in that moment of diagnoses, he or she loses a critical aspect of childhood.

In her book *Armfuls of Time,* Sourkes contends that “*the overwhelming nature of the illness cannot be approached by reality alone. Paradoxically, the illusion afforded by play is what allows reality to be integrated. Through play, the child can advance and retreat, draw near and pull away from the intense core. These tentative forays allow the child to contain and master the experience.*” She goes on to say that the fantastical world of the child at play should not be seen as avoidance of the truth because“*illusion is translucent, if not transparent*, *and thus reality shines through for both the child and the therapist even when not addressed directly by either*” [27] (p. 6).

Sourkes reminds us that children are not inclined to play when there is evidence of developmental disorders, severe psychopathology, or deprivation. It is also not unusual for the trauma of a life-threatening illness to temporarily extinguish the child’s capacity for play. However, within the context of psychotherapy, progress towards restoration can be seen when a child’s play returns to its former level of animation.

Thus, for children with life-limiting illnesses, or for children who have experienced trauma and stress as a result of a life-threatening diagnosis, play is the most natural means by which they can offload aggression, come to terms with the trauma of illness and impending death and attempt to take control of their world. Through play the child expresses traumatic fixations, conflicts and hostilities. The child also uses play to relax tension and anxiety [6].

### 6.3. Play Therapy in Children’s Palliative Care

“*Through play a child is able to release pent-up feelings of anxiety*,* disappointment*,* fear*,* aggression*,* insecurity*,* and confusion. Bringing these feelings to the surface encourages the child to deal with them*,* learn to master them*,* or abandon them. Through symbolic representation*,* the child gains a sense of control over events that seem uncontrollable in reality. Often*,* children are unable to verbally express what they are feeling*;* thus*, *in play therapy*, *toys serve as children’s words and play as their language.*”.

[40] (p. 240)

Whilst all children in palliative care have the need and right to play to express, explore and understand their experience and thoughts and feelings in relation to this, some children may additionally benefit from play therapy—a specific therapeutic intervention which uses play as the language through which the therapy takes place.

Based on a number of psychological theories, the use of play as a therapeutic intervention can be traced as far back as 1919. The works of Axline [41] and Oaklander [42] are probably the most significant to the growth of the discipline. There are two major approaches in play therapy, these being “Non-directive play therapy” and “Directive play therapy.” It is common for today’s practitioner to use a combination of both these approaches in accordance with the particular circumstances [43]. See Example 2. for Axline’s Eight Basic Principles of Non-Directive Play Therapy.

Play therapy can be defined as a specific counselling approach “*in which games, toys and mediums such as clay, drawings and paint are used to help a child or adolescent to express their emotions, thoughts, wishes and needs. It helps them to understand muddled feelings and upsetting events that they have not had the chance or the skills to sort out properly. Rather than having to explain what is troubling them, as adult therapy usually expects, children use play to communicate at their own level and at their own pace, without feeling interrogated or threatened*” [45]. Much of current play therapy practice is based upon the work of Virginia Axline [41], who is credited with being the inventor of non-directive play therapy.

In the 1974 publication of her book, “*Play Therapy*” Axline [41] asserts that there is a frankness, and honesty, and a vividness in the way children state themselves in a play situation. Their feelings, attitudes, and thoughts emerge, unfold themselves, twist and turn and lose their sharp edges. The child learns to understand himself and others a little better and to extend emotional hospitality to all people more generously. Bit by bit, with extreme caution, the child externalizes that inner self and states it with increasing candour and sometimes with dramatic flair. He soon learns that in this playroom with this unusual adult he can let in and out the tide of his feelings and impulses. He can create his own world with these simple toys that lend themselves so well to projected identities. He can be his own architect and create his castles in the sand, and he can people his world with the folks of his own making. He can select and discard. He can create and destroy. He can build himself a mountain and climb safely to the top and cry out for all his world to hear, “*I can build me a mountain or I can flatten it out. In here I am big*!” [41] (Preface).

**Box 2. Axline’s Eight Basic Principles of Non-Directive Play Therapy [42].**

The therapist:

- Must develop a warm and friendly relationship with the child.
- Accepts the child as she or he is.
- Establishes a feeling of permission in the relationship so that the child feels free to express his or her feelings completely.
- Is alert to recognise the feelings the child is expressing and reflects these feelings back in such a manner that the child gains insight into his/her behaviour.
- Maintains a deep respect for the child’s ability to solve his/her problems and gives the child the opportunity to do so. The responsibility to make choices and to institute change is the child’s.
- Does not attempt to direct the child’s actions or conversations in any manner. The child leads the way, the therapist follows.
- Does not hurry the therapy along. It is a gradual process and must be recognised as such by the therapist.
- Only establishes those limitations necessary to anchor the therapy to the world of reality and to make the child aware of his/her responsibility in the relationship.

(Source www.playtherapy.org.uk [44])

During non-directive play therapy, the therapist will behave in ways that will convey to the child the security and opportunity to explore not only the room and the toys but himself in this experience and relationship

Children communicate through words, body language and play [15,41] and Sourkes [27] contends that when children are given free rein to play, they are able to communicate their fears, hopes and allow observers to gain a deeper understanding of their situation. Those with an understanding of play therapy are able to use play as the medium to develop a therapeutic relationship with the child which in turn allows discussion about death to be a normal part of a child’s experience [36]. van Breeman cautions that learning to see and hear play should not be confused with playing with children. “*Children’s storytelling may occur using a sand tray*,* painting, dress-up*,* big-energy*,* drawing*,* and/or playing with archetypes. Children can express feelings about their family*,* how they view their illness*,* what they need to feel supported*,* how they want to live and who they need in their dying journey*” [36] (p. 512).

### 6.4. The Therapeutic Role of Play in Children’s Palliative Care

All children receiving palliative care need to play and whilst not all of them will need play therapy, the need for this type of therapeutic intervention will be higher within this more vulnerable population.

When considering the therapeutic role of play in children’s palliative care we are again reminded of the duality of these children who have a strong need to feel normal while their illness or condition imposes many additional needs upon them thus making them feel “different” and “abnormal”. Play has a role in responding to both the universal, but often overlooked, and the additional needs of these children. It does this by:

(a) Fulfilling the universal or basic needs of all children, including those in palliative care where the urge to play still exists. The role of play here is to support the developing mind of the child to learn about the world, to become competent at skills and master emotions. Play is a safe process in which the child takes control in expressing, exploring, experimenting and understanding their reality without failure and with the overall aim to master their experience.
(b) Fulfilling a specific psychosocial need existing in children in palliative care. The lived experience of a child in palliative care is characterised by stress, anxiety, obstacles to normal development, depression, fear, and loss of control, which in turn affects psychological, social and academic functioning. These children have additional needs to those of children not in palliative care such as the need for open and honest expression and communication with adults involved in their care, exploration and understanding of the dying process and involvement in end of life decisions.

While it is possible for unstructured play to adequately address both these needs, those who care for children in palliative care should be aware of when play therapy is indicated to allow a child the opportunity to express their fears and concerns.

The following case study from Sunflower Children’s Hospice in Bloemfontein, South Africa and written up on the ICPCN website [46], demonstrates the therapeutic value of play in the context of children’s palliative care.

*A young girl of 14 years who had a name that meant “Unwanted” in South Sotho was admitted to Sunflower House Children’s Hospice in Bloemfontein, South Africa. She was HIV infected and had advanced disease with severe malnutrition and depression. She did not make eye contact with the staff and sat outside on her own just gazing into space, unwilling to play with other children. Her family history showed she had been living with her grandmother after her mother died, and her father’s whereabouts were unknown. An occupational therapy student spent time with her trying to get her interested in games and activities, without success at first. Another girl of her age tried to interact with her, also without success. The psychologist reported that she suffered from a very poor self-image and felt worthless; and said she just was waiting to die.*

*The occupational therapist (OT) then brought along two dolls, dressed alike, and the child became interested in these dolls*,* enjoying dressing them in different clothing and playing with their hair. Eventually she started to make up a story—telling the OT that the one doll was called by her own name and identifying why she felt depressed, sad and lonely—and that she had never felt loved and wanted. The other doll she spoke of as the child she would like to be—happy*,* loved and with a dream for a future as a nurse. Over the course of two weeks*,* she gradually spent more time with the “happy” doll and eventually gave the other doll to another child. Her attitude changed to match that of the doll she played with—she responded to hugs and would sit on a staff member’s lap*;* she smiled and laughed with the other children*;* and she made friends with another young girl in the hospice. At the end she was very frail and could not keep any food down. Despite this, even on the day she died, she would insist on being amongst the other children in the garden*, *trying to eat whatever they ate*,* and she held her special doll in her hand*,* even when she lay dying. Needless to say, the doll was buried with her.*

### 6.5. Recommendations Regarding Play in Children’s Palliative Care

The following recommendations will allow the child to access play to fulfil their universal needs as children and their additional needs on account of their life limiting illness or condition.

- An encouragement for those supporting the child to not overlook the need and right of the ill child to play and therefore to not overlook opportunities, time and equipment needed to facilitate this play;
- An awareness and appreciation within health, social care and related professions of the importance of play for children in palliative care, and their potential role in facilitating play and listening to the child’s message as communicated through the play. Specific training for professionals regarding how to listen to the child through play could be useful here.
- Access to specialised play therapy interventions for those children who would benefit from such a provision.

## 7. Conclusions

A child receiving palliative care not only has the right but has an overwhelming need for time to be a child, to engage in childish pursuits such as developmentally appropriate play. The dying child needs activities that have no chance of failure as a way to balance the feelings of defeat and loss of mastery that comes with a diagnosis of a life-limiting illness. More than anything, these children need opportunities to communicate openly and honestly, to be enabled to express their very real fears or their feelings of anger. They need to know that there is someone on their care team that they can talk to about anything that concerns them, including about dying. Children without the necessary verbal skills, the maturity or the full comprehension of what they are experiencing only have the medium of play through which to communicate these anxieties and concerns. Those involved in their care need to have the skill to “listen” to what the child is telling them through their play and to respond appropriately.

The child with a life-limiting condition needs to be allowed as much independence and control as the illness allows. The loss of independence as the child’s body succumbs to an illness can cause withdrawal and depression. As play is self-chosen, self-directed and the child has the authority to stop at any chosen time, it is one of the few activities that provides the sick child with a sense of control and some liberation from any restrictions imposed upon them by their illness. The spiritual needs of the child and their need for reassurance that they are not alone in the dying process can be addressed through play and play therapy. Concerns about what will happen to them when they die, what will happen to their family and whether they will be missed, if not expressed verbally, may surface when a child is given the opportunity to participate in therapeutic play with carefully chosen toys and objects.

The role of play and play therapy to not only provide a pleasurable distraction and temporary respite from painful procedures and unpleasant experiences that accompany any life-threatening or life-limiting illness, is of inestimable value. Allowing the sick child the opportunity to escape from the real world with all its emotional trauma and painful procedures into a world of their own making, a fantasy world where they are the masters of their universe and they decide what magical powers they possess is a gift that only play can provide. However, the ability for the child to find a voice with which to express his or her innermost anxieties, fears and concerns and have these addressed makes the provision of a safe place to play, suitable toys to play with and regular play times in the presence of adult/s who have an understanding of play therapy and the therapeutic value of play vital elements in the delivery of good palliative care for children.
